# Epigenetic modulation of *Ceratorhiza hydrophila* by 5-azacytidine enhances antifungal metabolite production: insights from antimicrobial, metabolic, genomic and computational analyses

**DOI:** 10.1186/s12866-025-04330-8

**Published:** 2025-09-09

**Authors:** Rehab M. Abdelhamid, Elham R. S. Soliman, Eslam T. Mohamed, Yasmin M. Elsaba

**Affiliations:** Botany and Microbiology Department, Faculty of Science, Helwan University, Cairo, 11421 Egypt

**Keywords:** Epigenetic modulation, 5-azacytidine, *Ceratorhiza hydrophila*, Antimicrobial metabolites, GC-MS, Molecular docking, Biosynthetic pathways, Drug resistance.

## Abstract

**Background:**

The emergence of drug-resistant pathogens has stimulated the need for the development of new antimicrobial agents. Epigenetic modulation by suppressing epigenetic inhibitors, such as 5-azacytidine (5-aza), has been shown to activate silent biosynthetic gene clusters within a fungus and causes the production of novel secondary metabolites. This research examined this epigenetic modification strategy in the poorly studied filamentous fungus, *Ceratorhiza hydrophila*, which may help induce the additional production of bioactive compounds.

**Results:**

The results from genomic and spectroscopic analyses (ISSR profiling and FTIR spectroscopy) indicated that 50 µM 5-aza produced substantial global DNA demethylation and genomic changes in *C. hydrophila* with no impact on cell viability. The epigenetic changes associated with the DNA demethylation prompted a notable and selective change in antimicrobial profile to suppress antibacterial activity against strains such as *Clostridium sporogenes* while also showing a robust induction of antifungal activity against *Candida albicans* (22 mm inhibition zone). GC-MS was performed for a deep-dive characterization of the metabolic profile which revealed, for example, a dramatic alteration of the profile including production of new secondary metabolites such as a novel indole derivative and diisooctyl phthalate, which did not exist in the untreated control. In silico analyses, such as modelling the promoter and molecular docking opportunities, offered a believable mechanistic rationale for the effects seen, linked to the predicted modulation of primary biosynthetic pathways.

**Conclusion:**

This study demonstrates that epigenetic modulation can be used to successfully unlock latent biosynthetic capability in *C. hydrophila* resulting in the production of unique compounds with strong and selective antifungal activity. These results demonstrate the advantages of epigenetic screening of unique fungal sources in the search for new drug leads.

**Supplementary Information:**

The online version contains supplementary material available at 10.1186/s12866-025-04330-8.

## Introduction

The rise of pathogens that are resistant to multiple drugs is a major global health problem. To effectively fight infectious diseases, new antimicrobial agents need to be found [1]. Fungi are great at making secondary metabolites, which means they have a huge store of bioactive compounds that aren’t being used right now because of epigenetic regulation [2]. Epigenetic changes, like DNA methylation, are very important for changing how genes are expressed and how fungi make things. This is a promising way to unlock these silent gene clusters [3]. 5-azacytidine, a cytosine analog that stops DNA methyltransferases, is one of the most common epigenetic modulators used to change the metabolism of microorganisms. This leads to the creation of new bioactive metabolites that can kill bacteria [4].

*Ceratorhiza hydrophila* is a filamentous fungus that has been shown to have biosynthetic potential [5, 6]. It is a candidate for epigenetic manipulation that hasn’t been studied much yet. Previous research has shown that epigenetic changes in fungi can boost the production of antifungal and antibacterial compounds by turning on silent polyketide synthase (PKS) and fatty acid synthase (FAS) gene clusters [2]. But we still don’t know much about how 5-azacytidine affects the metabolism and antimicrobial properties of *C. hydrophila*.

The present investigation aimed to determine the effects of 5-azacytidine induced epigenetic modifications, and their effect on *C. hydrophila*. In our multi-faceted approach, we first employed genomic and spectroscopic analysis to validate the molecular mechanism by which 5-azacytidine was acting. We characterized the resulting changes to the fungus’ metabolic profile and its functional antimicrobial properties. We then took an integrated approach again and advanced a mechanistic hypothesis to describe the changes; utilizing a range of computational tools including promoter analysis and molecular docking. The exploration in this work demonstrated the use of concepts from a multi-faceted approach to unlock and characterize new and selective antimicrobial activities and novel secondary metabolites produced by an often unstudied aquatic fungus.

## Methods

### Induction of epigenetic mutations in *C. hydrophila* with 5-Azacytidine

*Ceratorhiza hydrophila* (accession no. MK387081), previously isolated from the aquatic plant *Myriophyllum spicatum* [5], was incubated in potato dextrose broth (PDB) cultures at 28 °C for a period of 7 days with shaking (150 rpm), and epigenetic modifications were induced on day 1 from 5-azacytidine (Sigma-Aldrich, Cat# A2385) at a final concentration of 50 µM put in the culture medium, this concentration was chosen based on previous studies examining its efficacy in inhibiting DNA methyltransferases in filamentous fungi whilst limiting the cytotoxicity of the compound [7]. A separate treatment-free control culture was grown under the same conditions.

After 7 days of incubation, the fungal cultures were harvested by centrifuging the culture flasks at 10,000x g for 10 min. The supernatant was collected and stored as cell-free filtrate. The remaining fungal hyphae that remained were washed twice with sterile PBS (pH 7.4) to remove any residual media, 5-azacytidine. The washed hyphae were then resuspended in 10 mL of sterile PBS and sonicated, on ice (Qsonica Q700 Sonicator), at 40% amplitude for 5 min (10s on, 20 s off cycle) to disintegrate the cells [8]. The sonicated suspension was then extracted three times with an equal volume of ethyl acetate (Sigma-Aldrich, Cat# 319902). The organic layers were combined and dried under a gentle air stream of nitrogen. The dry extract was then resuspended in 1mL of HPLC grade ethyl acetate for analysis. The remaining hyphal pellet was frozen and saved at −80 degrees Celcius for DNA extraction [9].

#### In vitro validation for the impact of 5-azacytidine on the DNA of *C. hydrophila*

### DNA extraction and genomic analyses

DNeasy^®^ Plant Mini Kit (QIAGEN, Cat# 1014630) was used to extract total genomic DNA from 100 mg (= 100 mg dry weight) of frozen hyphae of both untreated and treated cultures, using the protocol as outlined in the kit instructions. Quality and quantity of extracted DNA were confirmed by spectrophotometry (NanoDrop 2000) with all samples exhibiting A260/A280 ratio between 1.8 and 2.0.

### ISSR profiling

Inter-simple sequence repeats (ISSR) analysis was conducted to evaluate genomic modification. Five primers were used in the initial screening based on previous use and confirmed effectiveness in fungi genetics [10–14]. Subsequently, from the five primers screened, three primers with clear and repeatable bands, given *C. hydrophila* DNA, were included for analysis. PCR was performed as described in Saleh et al. [55] using 25 µL reactions consisting of 50 ng of genomic DNA, 1× PCR buffer, 2 mM MgCl₂, 200 µM dNTPs, 0.4 µM of primer, and 1 U of Taq DNA polymerase. The PCR products were resolved on a 1.5% agarose gel that was stained with ethidium bromide and visualized using UV light.

### Comet assay for DNA damage assessment

DNA damage was assessed using the single-cell gel electrophoresis (comet) assay [15, 16]. Nuclei were isolated from fungal hyphae, embedded in agarose on microscope slides, and lysed for 24 h at 4 °C. Following DNA unwinding in an alkaline buffer (pH 13), electrophoresis was conducted at 24 V for 30 min. The slides were neutralized, stained with ethidium bromide, and visualized with a fluorescence microscope. DNA damage parameters, including tail length, tail moment, and %DNA in the tail, were quantified for 50–150 nuclei per slide using CometScore v1.5 software [17].

### FTIR analysis of DNA from *C. hydrophila*

Differences in DNA conformation were analyzed using Fourier Transform Infrared (FTIR) spectroscopy (PerkinElmer FTIR Spectrometer). A 5 µL of eluted DNA ~ 50 ng/µL) was dropped onto the surface of diamond crystal of the ATR accessory. Spectra were collected from 4000 to 450 cm^−1^ at a resolution of 4 cm^−1^ and conducting 4 scans at a speed of 0.2 scans per second.

### Evaluation of the antimicrobial activity of cell and cell-free filtrate extracts of *C. hydrophila* treated with 5-azacytidine using the well diffusion method

The method of well diffusion, also known as agar diffusion, was used to evaluate the potential antimicrobial activity of both cell and cell free filtrate extracts from *C. hydrophila*. The extracts were tested on characterized Gram-positive, *Clostridium sporogenes* ATCC 3584, *Bacillus subtilis* ATCC 6633), Gram-negative, *Pseudomonas aeruginosa* ATCC 27853, *Klebsiella pneumoniae* ATCC 27736, *Salmonella enterica* ATCC 25566), and yeast, *Candida albicans* ATCC 10231 bacteria. Bacterial strains were grown on Mueller-Hinton Agar (MHA) while Sabouraud Dextrose Agar (SDA) was used for *C. albicans*, and all cultures were incubated at 37 °C for 24 h. *C. hydrophila* was grown in potato dextrose broth (PDB) medium for 7 days under shaking incubation at 28 °C (150 rpm). On the third day of incubation, *C. hydrophila* was treated with 5-azacytidine while keeping an untreated culture as a control. At the termination of the incubation period, fungal cells were collected through centrifugation (10,000 × g, 10 min) in which the supernatant served as the cell-free filtrate extract. The cell-free filtrate extract was filtered through a 0.22 μm membrane filter and extracted using ethyl acetate solvent. The cell extract was prepared by resuspending the fungal biomass with ethyl acetate and completing the extraction, followed by solvent evaporation for storage purposes prior to the completion of the antimicrobial tests. The antimicrobial activity was determined by placing 20 µL of each extract into wells with a diameter of 5 mm that had previously been inoculated with test microorganisms on agar plates. We utilized ciprofloxacin (5 µg/disc) as a positive control for tested bacteria; and Nystatin (100 µg/mL) for *C. albicans*. The plates were incubated at 37 °C for 24 h, we measured the inhibition zones in millimeters (mm) [39]. The experiments were conducted in triplicate and we indicated results as mean ± standard deviation (SD).

### GC-MS analysis of metabolic changes in *C. hydrophila* induced by 5-Azacytidine treatment

Gas Chromatography-Mass Spectrometry (GC-MS) was utilized to observe the metabolic profiles of the four extracts analyzed, using a Trace GC1310-ISQ Gas Chromatograph-Mass Spectrometer. The dried extracts underwent derivatization using trimethylsilyl (TMS) reagents to increase the volatility of the metabolites prior to injection. To determine the compounds, the mass spectra were compared to the NIST and Wiley databases.

### Bioinformatic analysis of biosynthetic genes and predictive modeling of epigenetic effects

A multi-step computational method was utilized to identify key biosynthetic genes, model cytosine demethylation effects, and predict changes in gene regulation and metabolic pathway activity.

#### Homology searches and gene identification

To identify key biosynthetic genes, the genome sequence of *C. hydrophila* (GenBank accession MK387081) was used in the NCBI BLASTn tool against the non-redundant nucleotide (nr/nt) database. We looked for sequences with significant homology to polyketide synthase (PKS) and fatty acid synthase (FAS) genes from other fungal organisms. We argued that the functions of these identified genes, Pks1 and Fas1, were putative because of their high percentage of nucleotide identity with the first BLAST hit from each gene: 67.2% identity with a PKS putative from *Aspergillus nidulans* and 69.8% identity with a FAS gene from *C. albicans*, respectively. We also used protein alignments to confirm functional domains were present in these putative genes using Clustal Omega.

#### In Silico demethylation and promoter analysis

The upstream promoter regions of the identified Pks1 and Fas1 genes and the relevant metabolic pathways of the other key genes were chosen for in silico demethylation analysis. This would provide evidence of the demethylating effects of 5-azacytidine. In the promoter sequences, all CpG dinucleotide locations were modified to TpG dinucleotides in order to simulate the demethylation by the 5-azacytidine, using EMBOSS Mutate. Each of these sequences was used as a “demethylated” sequence and were compared in their control (“original”) sequence condition. Both original and demethylated promoter sequences were analyzed for promoter strength using the iPromoter-2 L server. The results were reported as quantitative p-scores of predicted strength. In addition to comparing the p-scores for translational potential, the corresponding mRNA transcripts and the stability of those mRNAs were analyzed with RNAfold, a bioinformatics tool that calculates the free energy (ΔG) of the predicted mRNA secondary structure.

#### Modeling pathways

To assess the wider impacts on metabolism, we modeled the downstream metabolic impacts of demethylation on Phenylpropanoid Biosynthesis (KEGG map00940) and Terpenoid Backbone Biosynthesis (KEGG map00900) pathways. The expression fold-changes for relevant genes in these pathways (e.g. f3h, chs, ters) reported in the results were found computationally by relating the extent of the deviation in predictive p-score from iPromoter-2 L between the control and the “demethylated” promoter sequence. By providing an in silico model whereby we could make a reasonable estimate of their up- or down-regulation; we were able to provide a systems-level understanding of the metabolic reprogramming effect resulting from 5-azacytidine.

### Computational and experimental insights into the antimicrobial potential of bioactive compounds: molecular docking study against bacterial and fungal targets

Using the predicted molecular docking and the Glide module in Maestro Schrödinger (v. 13.4), a mechanistic hypothesis of the reported antibacterial mechanism of action was developed. The three-dimensional crystal structures of four essential microbial enzymes were identified as receptors from the Protein Data Bank, including *B. subtilis* Transferase (2W8D), an enzyme associated with cell wall biosynthesis [18]; *S. enterica* Transferase (7KGN), which is also required for bacterial cell wall synthesis [19]; *C. albicans* CYP51 (5V5Z), a critical enzyme for ergosterol biosynthesis and fungal membrane integrity [20]; and *K. pneumoniae* FabG (6T77), a key enzyme in fatty acid biosynthesis for membrane construction [20]. These proteins are selected as validated drug-target pathways needed for microbial survival such as cell wall, membrane and ergosterol biosynthesis. Each receptor structure will be generated using the Protein Preparation Wizard, including optimization, reductions in energy and removal of unnecessary water.

Ligands for the study included the bioactive compounds identified via GC-MS. The standard drugs ciprofloxacin and nystatin were also included for comparative benchmarking, consistent with the in vitro assays. All ligand structures were prepared using LigPrep to generate stable, low-energy 3D conformers. Docking was performed in standard precision (SP) mode using a grid-based method, with the grid box centered on the known active site of each protein. The binding affinity for each ligand-receptor pair was evaluated based on the calculated docking score, where a more negative score indicates a stronger predicted interaction. The resulting poses were analyzed to elucidate potential structure-activity relationships [21].

### Computational ADME profiling of bioactive metabolites identified in *C. hydrophila*: an in silico pharmacokinetic assessment

In silico Absorption, Distribution, Metabolism, and Excretion (ADME) analysis was conducted using the Schrödinger QikProp module to assess the pharmacokinetic and drug-like properties of selected metabolites based on selected descriptors including molecular weight (MW), lipophilicity (QPlogPo/w) and aqueous solubility (QPlogS) [22].

### Gene interaction analysis for targeting bacterial cell wall and *Candida* ergosterol pathways: a computational approach

To validate the importance of the chosen drug-target pathways, gene interaction networks were created with GeneMANIA. The network consisted of genes implicated in peptidoglycan biosynthesis in bacteria and ergosterol metabolism in *C. albicans* to visualize the interconnectedness of these fundamental pathways [23].

## Results

### In vitro validation for the impact of 5-Azacytidine on the DNA of *C. hydrophila*

#### Genetic profiling using ISSR markers

**Fig. 1:**
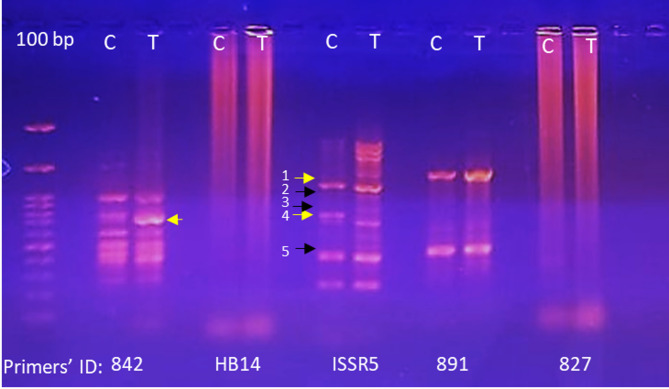
Agarose gel electrophoresis showing ISSR marker profiles of *C. hydrophila* genomic DNA in response to 5-azacytidine treatment. Yellow arrows indicate band presence, black arrows indicate band absence, and 100 bp refers to the DNA ladder

Inter-Simple Sequence Repeat (ISSR) analysis revealed genomic changes in *C. hydrophila* induced by 5-azacytidine. Three of five available (I-842, I-891 and ISSR-5) produced bands that were detectable, consistent and reproducible (Table 1). The results of 5-azacytidine selected numerous changes to the banding patterns including novel bands (700 bp band confirmed that with the primer I-842) and missing bands (several bands observed in the untreated control were absent) (Fig. 1). Collectively these results suggested that 5-azacytidine had sequence specific effects on genomic loci at treatment.

**Table 1:** ISSR primers used for genomic profiling of *C. hydrophila* in response to 5-azacytidine treatment, including primer sequences, total bands amplified, and size range

Primer code	Sequence (5´→3´)			Total bands amplified	Size range (bp)	
I-842	(GA)⁸CTG			7	2000 − 250	
I-891	ACTACGACT(TG)_5_T			4	1900 − 410	
ISSR-5	(ACG)⁴GAC			13	2600 − 220	
HB14	(GT)_6_CC			-	-	
I-827	(AC)_8_G			-	-	
Total number of markers			21			

### Assessment of DNA damage using comet assay

**Fig. 2:**
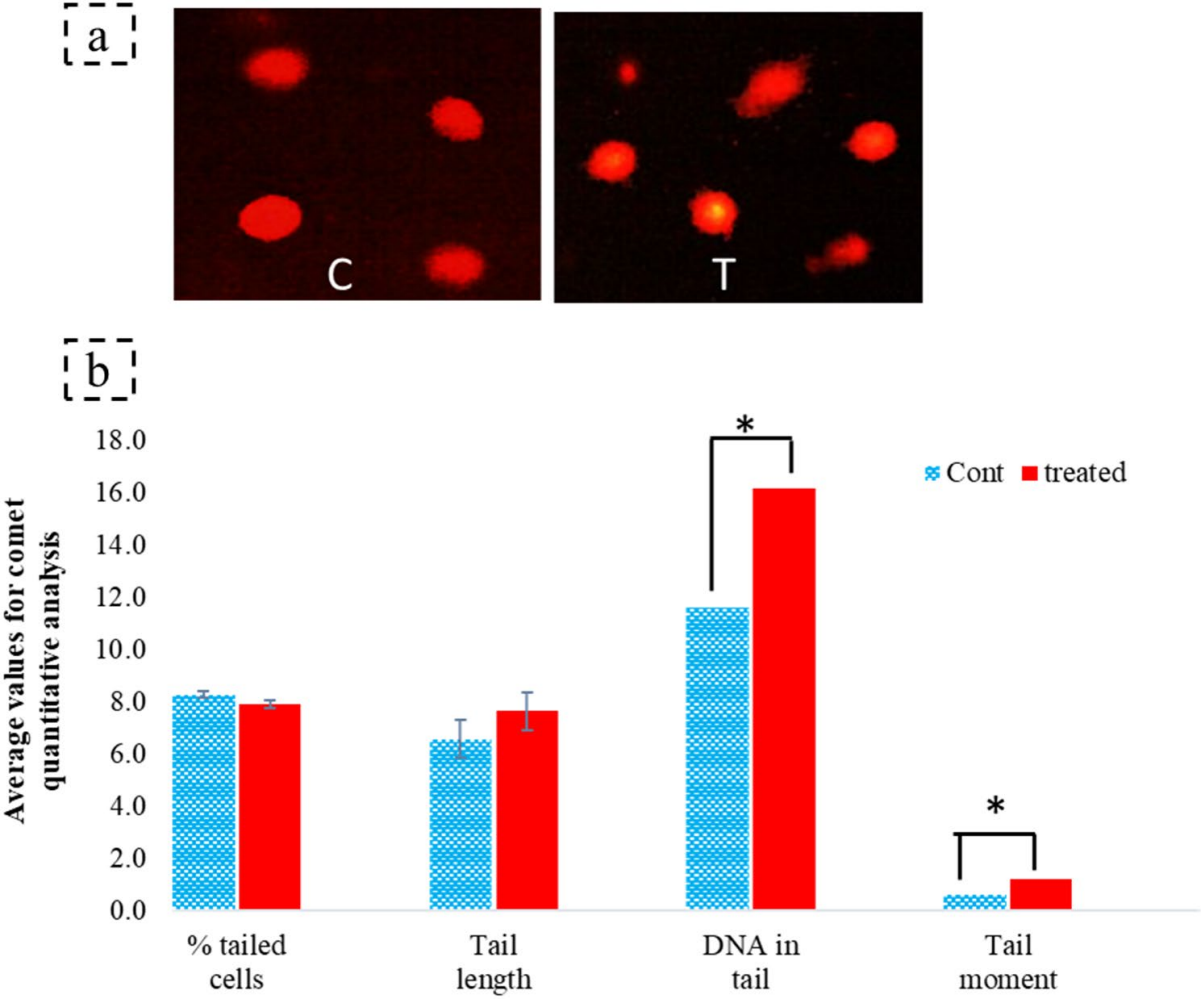
Comet assay analysis of DNA damage in *C. hydrophila* following 5-azacytidine treatment. (**a**) qualitative visualization of nuclei from control (C) and treated (T) samples. (**b**) quantitative analysis of DNA damage parameters, including percentage of DNA in tail and tail moment. Asterisk (*) denotes statistically significant differences (t-test, *n* = 6, *p* < 0.05)

The comet assay was utilized to measure the DNA damage. Overall, the treated and control samples did not differ in the total percentage of damaged cells, yet the treatment did have an effect on cells that already had DNA breaks (Fig. 2). In these cases, treatment with 5-azacytidine yielded a statistically significant (*p* < 0.05) increase in both the percentage of DNA in the tail (39.3%) and the tail moment (95.13%). The results suggested an amplification of damage to cells that were previously compromised.

### Effect of 5-azacytidine on DNA conformation and functional groups

**Fig. 3:**
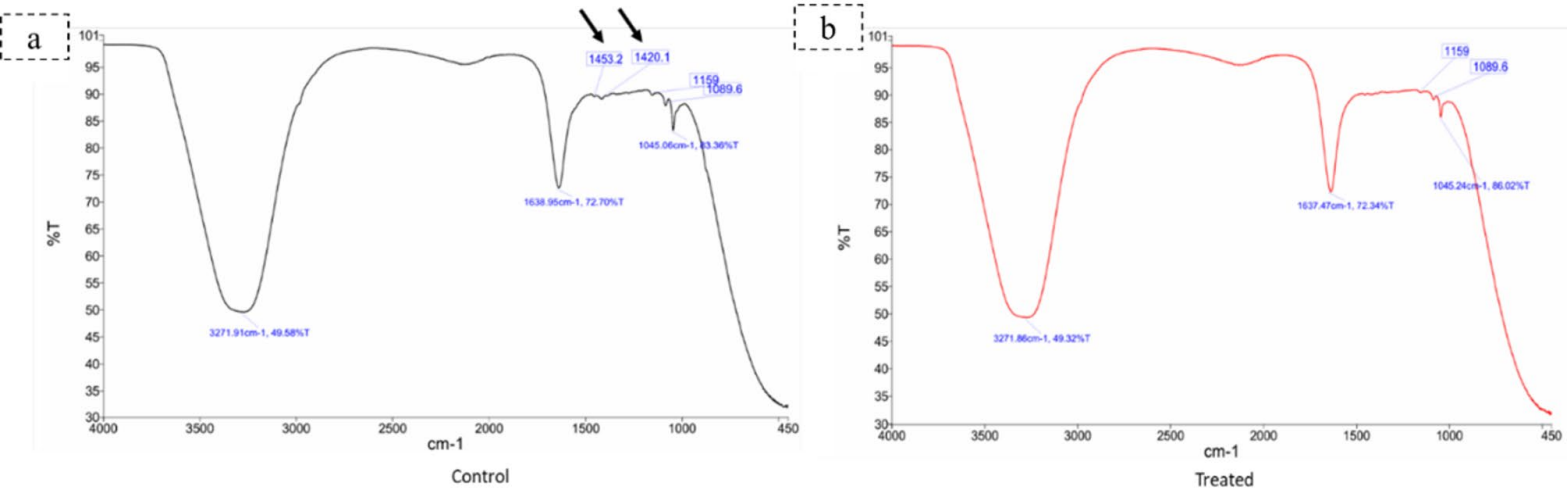
FTIR spectra of DNA from (**a**) control and (**b**) 5-azacytidine-treated *C. hydrophila*. While the main backbone peaks remain, the key evidence of epigenetic modification is the disappearance of the peaks at 1453 cm⁻¹ and 1420 cm⁻¹ in the treated sample (highlighted by arrows in panel ‘a’ and their absence in ‘b’), indicating successful demethylation and structural alteration

Fourier Transform Infrared (FTIR) spectroscopy provided direct evidence of epigenetic modification. While the core structure of the DNA backbone was maintained in both samples, the DNA from the treated fungus showed a complete absence of the spectral peaks at 1420 cm⁻¹ and 1453 cm⁻¹ (Table 2; Fig. 3). The disappearance of these peaks, which are associated with B-form DNA base stacking and CH₂ vibrations linked to DNA methylation, implies successful demethylation and a resulting alteration in DNA structure.

**Table 2:** FTIR spectral peaks and corresponding functional groups in DNA of *C. hydrophila* in response to 5-azacytidine treatment

Frequencies (wave length (cm^−1^)	Comment	Presence of bands	
		Control (non-5-azacytidine treated sample)	5-azacytidine treated sample
3271.86	OH and NH bonds of the amino acid	√	√
1638.95	C = C thymine, adenine vibrations, and N − H from guanine	√	√
1453.2	CH₂ scissoring vibrations and methylene groups in the DNA	√	
1420.1	Base/in-plane vibration	√	
1159	C-O and C-C stretching vibrations of the deoxyribose sugar	√	√
1089.6	Backbone- phosphate symmetric stretching PO2	√	√
1045.06	C − O deoxyribose stretching	√	√

### Evaluation of the antimicrobial activity of cell and cell-free filtrate extracts of *C. hydrophila* treated with 5-azacytidine using the well diffusion method

**Fig. 4:**
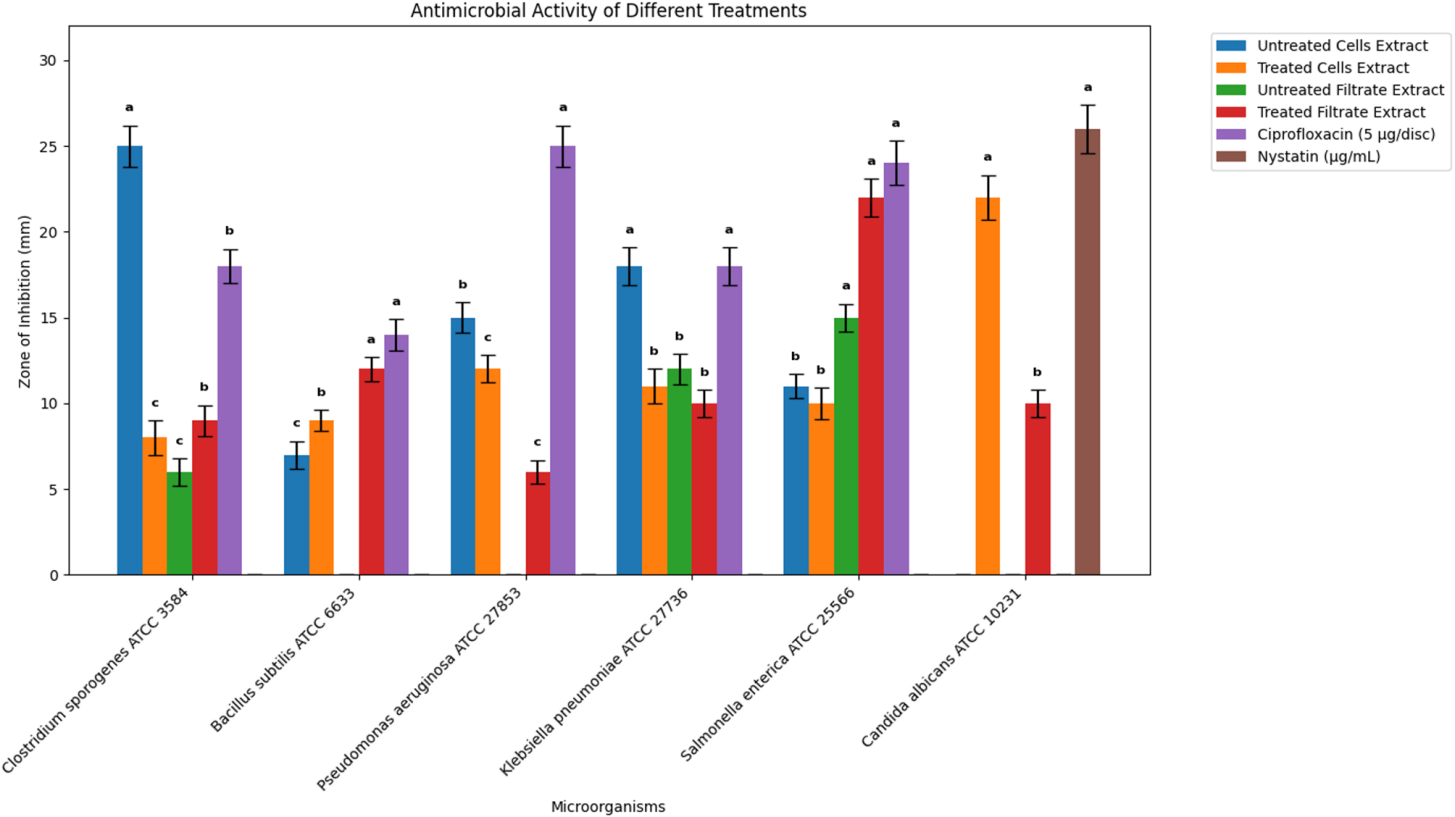
Inhibition zone (mm) of extracts against microbial strains. Values are presented as mean ± SD (*n* = 3). Different superscript letters (ᵃ, ᵇ, ᶜ) within a column indicate statistically significant differences (*p* < 0.05)

**Fig. 5:**
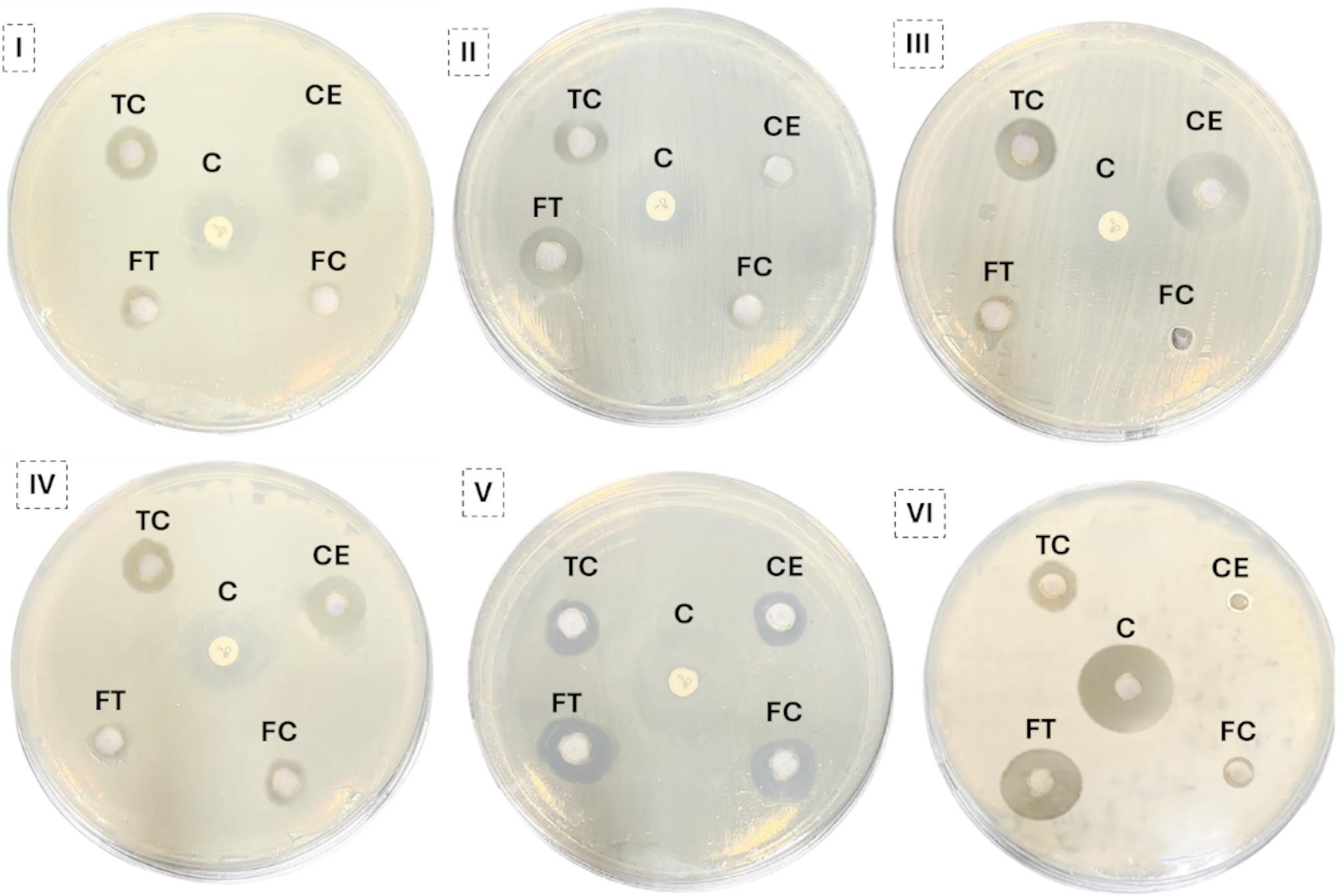
Visual representation of the antimicrobial activity of *C. hydrophila* extracts, providing qualitative support for the quantitative data in Fig. 4. Note the contrasting effects of the treatment, such as the general reduction in antibacterial activity versus the enhanced antifungal activity against *C. albicans*. Test microorganisms: (**I**) *C. sporogenes*, (**II**) *B. subtilis*, (**III**) *P. aeruginosa*, (**IV**) *K. pneumoniae*, (**V**) *S. enterica*, and (**VI**) *C. albicans*. Abbreviations: (CE) Untreated cells extract; (TC) Treated cells extract; (FC) Untreated cell filtrate extract; (FT) Treated cell filtrate extract. The positive control (C) was ciprofloxacin (5 µg/disc) for bacteria and nystatin (100 µg/ml) for fungi

**Fig. 6:**
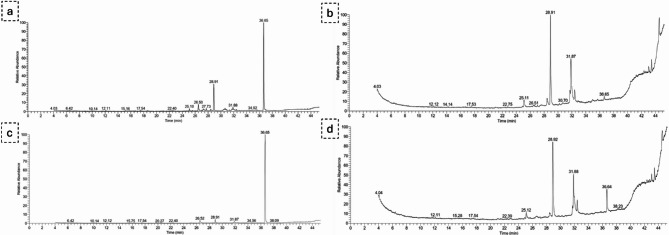
GC-MS analysis of *C. hydrophila* treatments: (**a**) untreated cells extract, (**b**) treated cells extract, (**c**) untreated cell filtrate extract, (**d**) treated cell filtrate extract

The extracts from treated and untreated *C. hydrophila* showed significantly different antimicrobial profiles (Figs. 4 and 5). The untreated cell extract was most active against *C. sporogenes* (25 mm inhibition zone). Following treatment, this activity was significantly reduced (8 mm). Conversely, the treated cell extract demonstrated a potent, newly acquired antifungal activity against *C. albicans* (22 mm). On the other hand , the treated filtrate extract showed enhanced activity against *S. enterica* (22 mm) and *B. subtilis* (12 mm). These results indicate a clear, selective reprogramming of the fungus’s antimicrobial output (Fig. 6).

### GC-MS analysis of metabolic changes in *C. hydrophila* induced by 5-Azacytidine treatment

The results of GC-MS analysis comparing the untreated and 5-azacytidine-treated extracts of *C. hydrophila* revealed notable differences in the composition of metabolites. In the untreated cell extracts, most of the metabolites were myristic acid, palmitoleic acid, hexadecanoic acid, and oleic acid derivatives; all of these compounds have been reported to have antimicrobial, antioxidant, and anti-inflammatory properties. The metabolites dibutyl phthalate (DBP) and bis(2-ethylhexyl) phthalate (DEHP) were detected in *C. hydrophila* untreated samples; it is worthy of mention that DBP and DEHP exhibit weak antimicrobial activity [ 24]. The natural occurrence of the compound 9,12-octadecadienoic acid (Z, Z) or octadecadienoic acid in the untreated samples could indicate moderate antibacterial and antifungal activity [25]. After the introduction of 5-azacytidine metabolites appeared in the extracts that were not seen in the untreated extracts. Of the novel metabolites, we identified the compound 2-acetyl-3-(2-cinnamido)ethyl-7-methoxyindole; this is a novel indole derivative with potential antimicrobial and antioxidant properties [26]. Furthermore, all the extracts induced the expression of at least greater abundances of palmitoleic acid, hexadecanoic acid and octadecanoic acid, these compounds demonstrate some antibacterial effects [27–30]. Along with, for those treated filtrates also had 10-undecenoic acid which indicates better antibacterial and antifungal activity [31]. In conclusion these results show promise for 5-azacytidine as an agent for activating silent biosynthetic pathways, the production of novel secondary metabolites that show potential application for antimicrobial tasks (Table 3; Fig. 2).

**Table 3:** Metabolites identified by GC-MS in untreated and treated *C. hydrophila* extracts with their corresponding spectral match factor (MF) and reported biological activities

Sample type	Compound name (TMS derivative)	Retention Time (min)	Molecular structure	Molecular weight (g/mol)	Match factor (MF)	Biological activity
Untreated cells extract	Myristic acid, TMS derivative	3.80	CH₃(CH₂)₁₂COOSi(CH₃)₃	286	883	Antimicrobial, Antioxidant, Anti-inflammatory [33].
	Dibutyl phthalate	7.50	C₆H₄(COOCH₂CH₂CH₂CH₃)₂	278	935	Antimicrobial, Antioxidant [25].
	n-Hexadecanoic acid	8.90	CH₃(CH₂)₇CH = CH(CH₂)₇COOH	282	824	Antimicrobial, Antioxidant, Anti-inflammatory [33].
	Hexadecanoic acid, ethyl ester	6.10	CH₃(CH₂)₁₄COOCH₂CH₃	284	860	Antimicrobial, Antioxidant [28].
	Palmitoleic acid, TMS derivative	5.40	CH₃(CH₂)₅CH = CH(CH₂)₇COOSi(CH₃)₃	326	812	Antimicrobial, Antioxidant, Anti-diabetic [27].
	Hexadecanoic acid, TMS derivative	6.50	CH₃(CH₂)₁₄COOSi(CH₃)₃	328	937	Antimicrobial, Antioxidant, Anticancer [29].
	9,12-Octadecadienoyl chloride, (Z, Z)-	8.70	CH₃(CH₂)₄CH = CHCH₂CH = CH(CH₂)₇COCl	296	858	Antimicrobial, Antioxidant [34].
	9,12-Octadecadienoic acid (Z, Z)-, TMS derivative	9.10	CH₃(CH₂)₄CH = CHCH₂CH = CH(CH₂)₇COOSi(CH₃)₃	352	855	Antimicrobial, Antioxidant, Anti-inflammatory [25].
	Oleic Acid, (Z)-, TMS derivative	9.30	CH₃(CH₂)₇CH = CH(CH₂)₇COOSi(CH₃)₃	354	886	Antimicrobial, Antioxidant, Anti-inflammatory [35].
	Bis(2-ethylhexyl) phthalate	10.50	C₆H₄[COO-CH₂CH(C₂H₅)(CH₂)₃CH₃]₂	390	954	Antimicrobial, Antioxidant [24].
Treated cells extract	Myristic acid, TMS derivative	5.45	CH₃(CH₂)₅CH = CH(CH₂)₇COOSi(CH₃)₃	326	775	Antimicrobial, Antioxidant, Anti-diabetic [27].
	Palmitoleic acid, TMS derivative	6.55	CH₃(CH₂)₁₄COOSi(CH₃)₃	328	802	Antimicrobial, Antioxidant, Anticancer [29].
	Hexadecanoic acid, TMS derivative	9.15	CH₃(CH₂)₄CH = CHCH₂CH = CH(CH₂)₇COOSi(CH₃)₃	352	892	Antimicrobial, Antioxidant, Anti-inflammatory [25].
	9,12-Octadecadienoic acid (Z, Z)-, TMS derivative	9.35	CH₃(CH₂)₇CH = CH(CH₂)₇COOSi(CH₃)₃	354	844	Antimicrobial, Antioxidant, Anti-inflammatory [35].
	Oleic Acid, (Z)-, TMS derivative	9.70	CH₃(CH₂)₁₆COOSi(CH₃)₃	356	831	Antimicrobial, Antioxidant, Anti-inflammatory [30].
	Octadecanoic acid, TMS derivative	11.20	CCNC_1_ = NC(= NC(= N_1_)N)Cl	350	809	Antimicrobial, Antioxidant, Anticancer [26].
Untreated cell filtrate extract	2-Acetyl-3-(2-cinnamido)ethyl-7-methoxyindole	7.55	C₆H₄(COOCH₂CH₂CH₂CH₃)₂	278	682	Antimicrobial, Antioxidant [36].
	1,2-Benzenedicarboxylic acid, dibutyl ester	6.60	CH₃(CH₂)₁₄COOSi(CH₃)₃	328	944	Antimicrobial, Antioxidant, Anticancer [29].
	Hexadecanoic acid, TMS derivative	9.40	CH₃(CH₂)₇CH = CH(CH₂)₇COOSi(CH₃)₃	354	896	Antimicrobial, Antioxidant, Anti-inflammatory [35].
	Oleic Acid, (Z)-, TMS derivative	10.55	C₆H₄[COO-CH₂CH(C₂H₅)(CH₂)₃CH₃]₂	390	867	Antimicrobial, Antioxidant (Javed et al., 2022).
Treated cell filtrate extract	Diisooctyl phthalate (Bis(2-ethylhexyl) phthalate)	4.20	CH₂=CH(CH₂)₈COOSi(CH₃)₃	270	945	Antimicrobial, Antioxidant, Antiviral [31].
	10-Undecenoic acid, TMS derivative	5.50	CH₃(CH₂)₅CH = CH(CH₂)₇COOSi(CH₃)₃	326	793	Antimicrobial, Antioxidant, Anti-diabetic [27].
	Palmitoleic acid, TMS derivative	6.65	CH₃(CH₂)₁₄COOSi(CH₃)₃	328	788	Antimicrobial, Antioxidant, Anticancer [29].
	Hexadecanoic acid, TMS derivative	9.20	CH₃(CH₂)₄CH = CHCH₂CH = CH(CH₂)₇COOSi(CH₃)₃	352	916	Antimicrobial, Antioxidant, Anti-inflammatory [25].
	9,12-Octadecadienoic acid (Z, Z)-, TMS derivative	9.45	CH₃(CH₂)₇CH = CH(CH₂)₇COOSi(CH₃)₃	354	812	Antimicrobial, Antioxidant, Anti-inflammatory [35].
	Oleic Acid, (Z)-, TMS derivative	9.75	CH₃(CH₂)₁₆COOSi(CH₃)₃	356	847	Antimicrobial, Antioxidant, Anti-inflammatory [30].
	Octadecanoic acid, TMS derivative	10.60	C₆H₄[COO-CH₂CH(CH₃)(CH₂)₄CH₃]₂	390	809	Antimicrobial, Antioxidant [32].

### Bioinformatic analysis of biosynthetic genes and predictive modeling of epigenetic effects

**Fig. 7:**
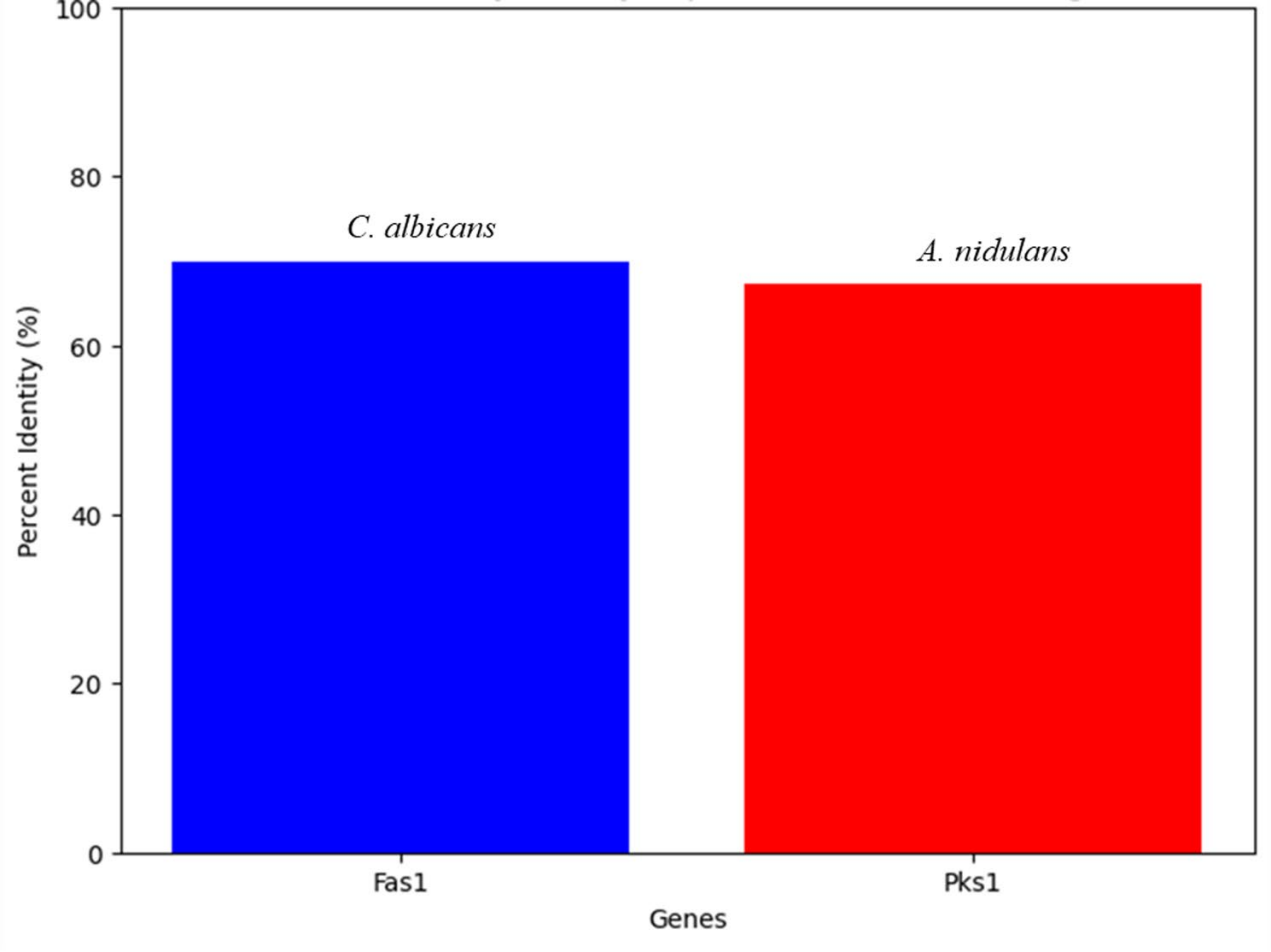
Percent identity of *C. hydrophila* genes with their homologs: *Fas1* vs. *C. albicans* (69.8%) and *Pks1* vs. *A. nidulans* (67.2%) based on NCBI BLASTn analysis

**Fig. 8:**
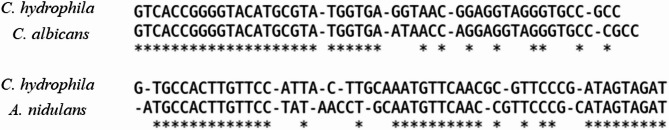
Sequence alignments of *Fas1* and *Pks1* promoters before and after simulated demethylation, showing variations in CpG-rich regions that influence transcription factor binding and promoter activity

Considering the substantial treatment-induced variations to the fatty acid and secondary metabolite profiles identified via GC-MS, along with the associated shift in antimicrobial activity, we directed our computational analysis toward the promoters of the fatty acid synthase (Fas1) and polyketide synthase (Pks1) genes as they are the most relevant biosynthetic pathways related to our experimental work. The study team used NCBI BLASTn to uncover many gene clusters homologous to fungal polyketide synthase (PKS) genes and fatty acid synthase (FAS) genes. Pks1 exhibited a 67.2% identity with *Aspergillus nidulans* PKS. Fas1 exhibited a 69.8% identity with *C. albicans* FAS. The data are encapsulated in Fig. 7, and further sequence alignments (Fig. 8) demonstrated that the genes were all highly conserved.

The effect of 5-azacytidine on the transcriptional activity of the promoters and the stability of the mRNAs were analyzed by simulating demethylation with EMBOSS Mutate. Clustal Omega alignments showed that there were slight differences in terms of sequence in the GC rich areas which had possible effects on the promoter activity. The Fas1 promoter increased the predicted activity from its p-score of 0.62 to a p-score of 0.89, whereas the Pks1 promoter decreased p-score wise from 0.75 to 0.41. Overall, the changes in predicted activity related to differences in transcriptional regulations were plausible, and were also confirmed based on iPromoter-2 L predictions (as noted in Fig. S1). Further, RNA fold analysis showed there were differences in the stability of the mRNA but Fas1 stability improved with free energy shifting from − 32.5 kcal/mol to −38.7 kcal/mol suggesting a possible improvement in translational efficiency. On the other hand, RNAfold analysis indicated that mRNA stability for Fas1 did increase (ΔG from − 32.5 kcal/mol to −38.7 kcal/mol) suggesting improvement in translational efficiency while Pks1 mRNA stability decreased (ΔG from − 35.2 kcal/mol to −29.8 kcal/mol) (Fig. S2).

**Fig. 9:**
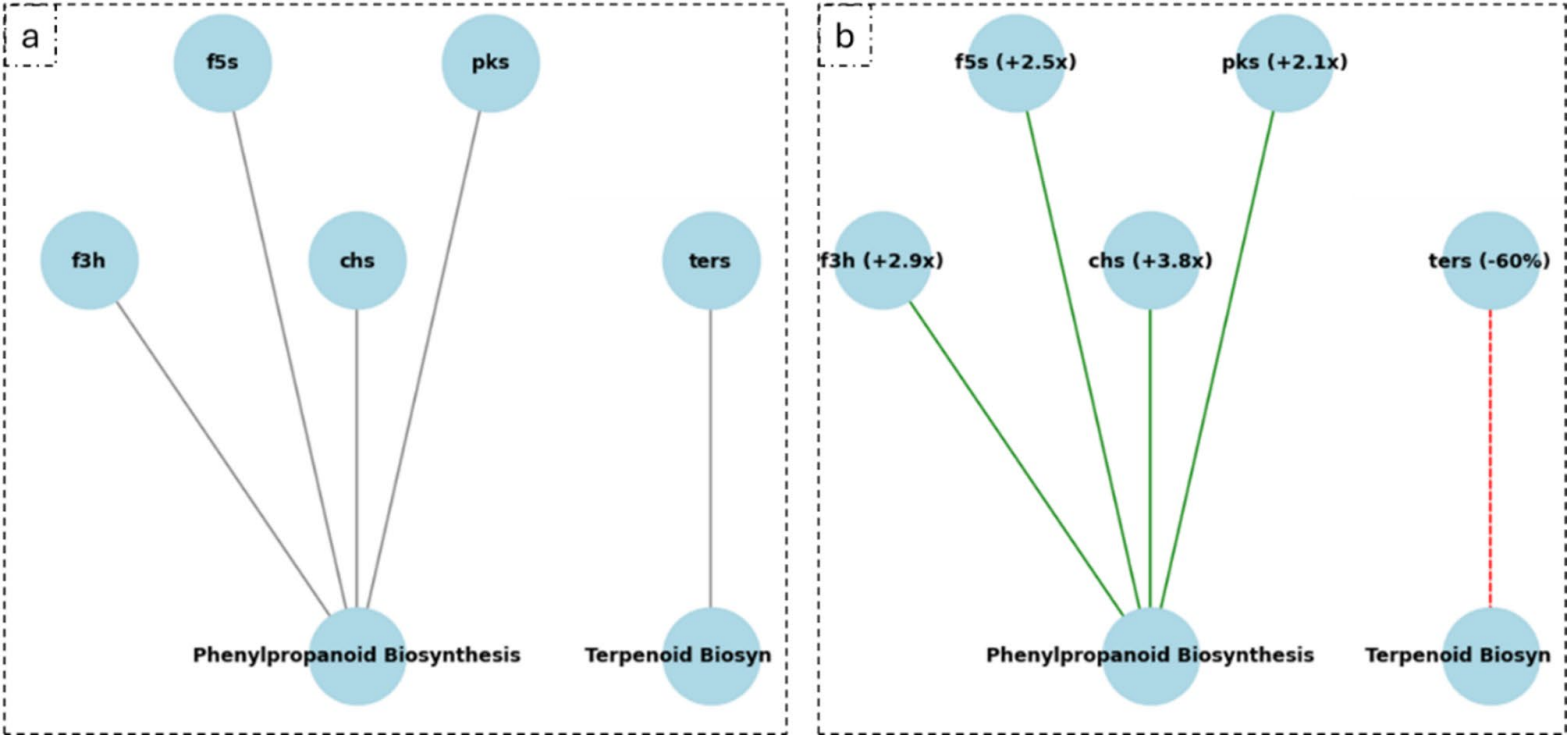
KEGG pathway diagrams show metabolic pathways in (**a**) untreated *C. hydrophila* and (**b**) 5-azacytidine-treated treatment, highlighting activation of phenylpropanoid biosynthesis and suppression of terpenoid biosynthesis

The KEGG pathway analysis demonstrated the notable metabolic changes that occurred as a result of 5-azacytidine treatment (Table 3). From the KEGG pathway analysis, phenylpropanoid biosynthetic pathways were highly up-regulated (increased the expression of f3h (2.9-fold), f5s (2.5-fold), chs (3.8-fold), and pks (2.1-fold) from pathways), whereas the terpenoid biosynthesis pathways activity was noticeably down-regulated, resulting in a reduction of ters expression of 60% (Fig. 9). These altered metabolic analyses coincided with the increased antifungal activity and reduced antibacterial activity in the extracts. The KEGG mapping result is summarized in Fig. 9.

### Computational and experimental insights into the antimicrobial potential of bioactive compounds: molecular docking study against bacterial and fungal targets

**Fig. 10:**
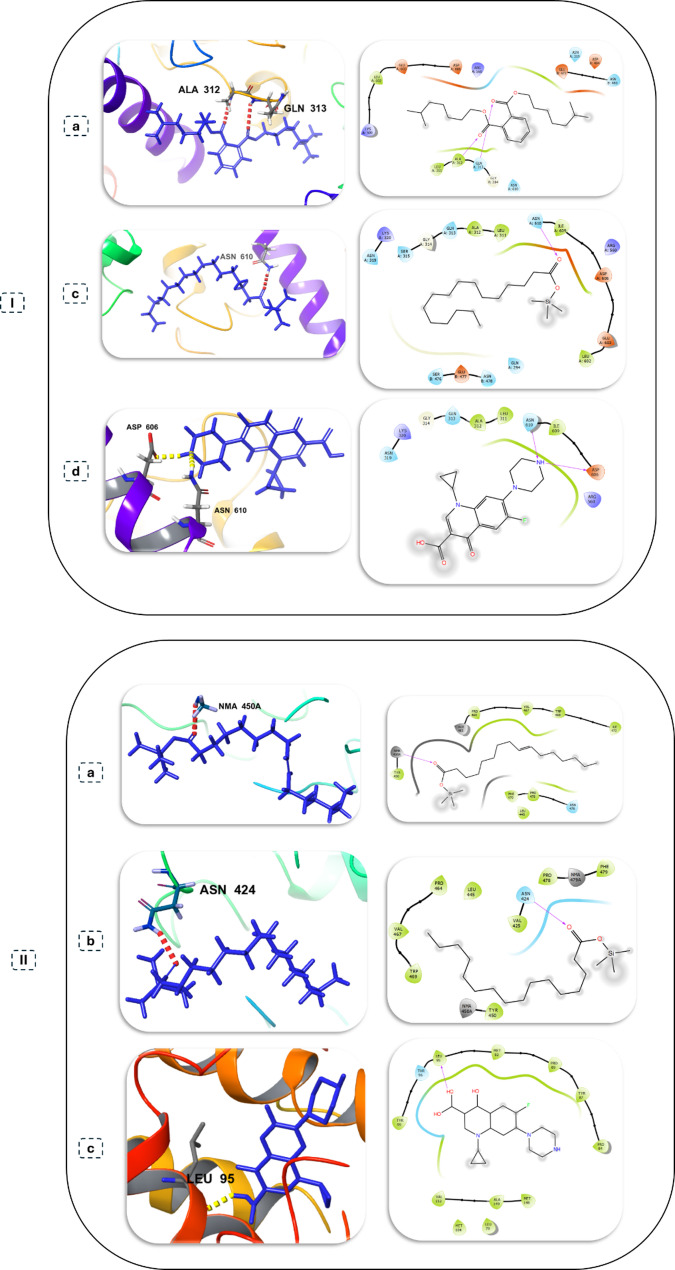
Molecular docking analysis of identified metabolites with key microbial enzymes. Each panel displays the 3D docked pose alongside its corresponding 2D ligand interaction diagram of: (**I**) *B. subtilis*, transferase, cell wall synthesis with (**a**) Diisooctyl phthalate, (**b**) Hexadecanoic acid, TMS derivative, (**c**) ciprofloxacin and ( **II**) *S. enterica*, transferase, cell wall synthesis with (**a**) Palmitoleic acid, TMS derivative, (**b**) Hexadecanoic acid, TMS derivative, (**c**) ciprofloxacin

**Fig. 11:**
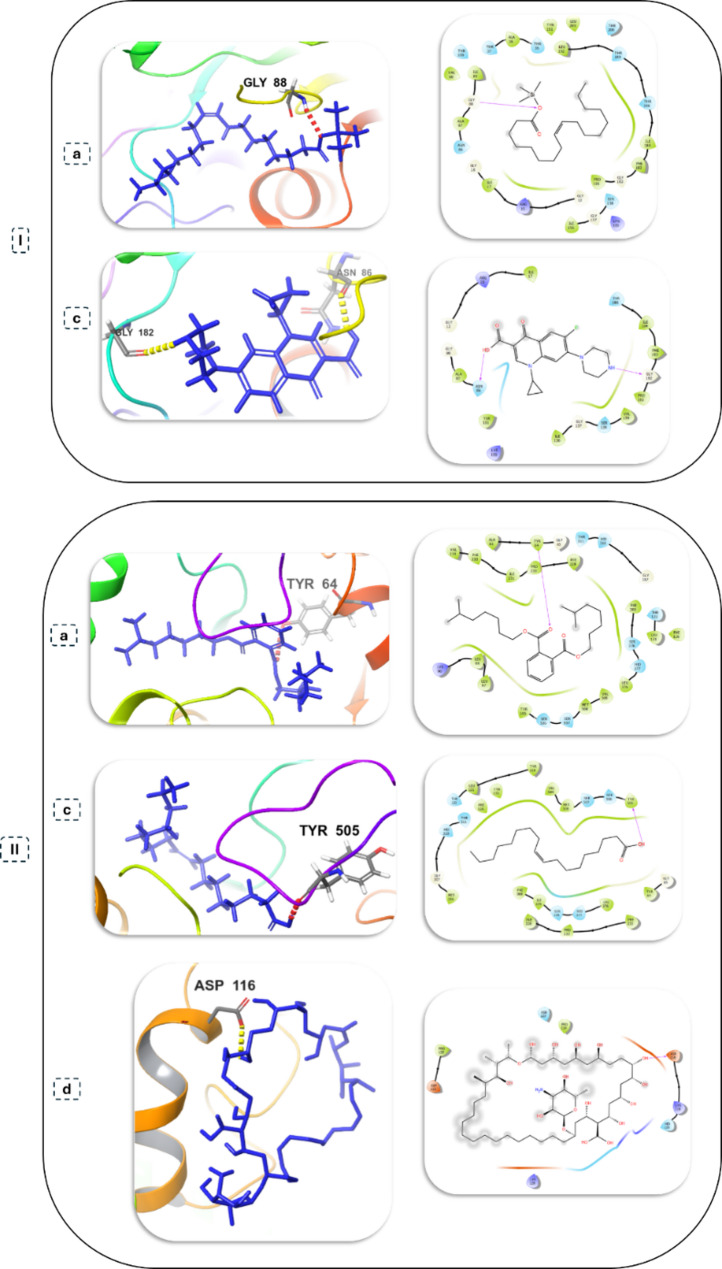
Molecular docking analysis of identified metabolites with key microbial enzymes. Each panel displays the 3D docked pose alongside its corresponding 2D ligand interaction diagram of: (**I**) *K. pneumoniae*, FabG, membrane synthesis with (**a**) Oleic Acid (Z)-, TMS derivative, (**b**) ciprofloxacin and (**II**) *C. albicans*, CYP51, oxidoreductase, ergosterol synthesis with (**a**) Diisooctyl phthalate, (**b**) 9-Octadecenoic acid, (**d**) nystatin

A predictive molecular docking study was conducted to provide a mechanistic hypothesis for the changed antimicrobial activity. This study assessed the binding affinity of the identified metabolites to critical microbial enzymes (Table 4). Extracts from the treated samples had the best binding affinities with metabolites, with the strongest being diisooctyl phthalate to *C. albicans* CYP51 (−4.767 kcal/mol) and oleic acid to *K. pneumoniae* FabG (−4.111 kcal/mol). These results demonstrate a potential molecular basis for the improved antifungal activity and selective antibacterial activity observed in the treated samples (Figs. 10 and 11).

**Table 4:** Compounds from each extract with the highest Docking scores against microbial target proteins

Microbial target proteins	Sample type	Compound	Highest docking score (kcal/mol)	H-bond
*Bacillus subtilis* (Transferase, Cell wall synthesis)	Untreated cells extract	Hexadecanoic acid, TMS derivative	−1.824	ASN 610
	Treated cells extract	Hexadecanoic acid, TMS derivative	−1.824	ASN 610
	Untreated filtrate extract	Hexadecanoic acid, TMS derivative	−1.824	ASN 610
	Treated filtrate extract	Diisooctyl phthalate	−2.299	ALA 312, GLN 313
	Standard drug	Ciprofloxacin	−3.934	ASP 606, ASN 610
*Salmonella enterica* (Transferase, Cell wall synthesis)	Untreated cells extract	Palmitoleic acid, TMS derivative	−3.393	NMA 350
	Treated cells extract	Hexadecanoic acid, TMS derivative	−2.055	ASN 424
	Untreated filtrate extract	Hexadecanoic acid, TMS derivative	−2.055	ASN 424
	Treated filtrate extract	Hexadecanoic acid, TMS derivative	−2.055	ASN 424
	Standard drug	Ciprofloxacin	−4.185	LEU 95
*Klebsiella pneumoniae* (FabG, Membrane synthesis)	Untreated cells extract	Oleic Acid, (Z)-, TMS derivative	−4.111	GLY 88
	Treated cells extract	Oleic Acid, (Z)-, TMS derivative	−4.111	GLY 88
	Untreated filtrate extract	Oleic Acid, (Z)-, TMS derivative	−4.111	GLY 88
	Treated filtrate extract	Oleic Acid, (Z)-, TMS derivative	−4.111	GLY 88
	Standard drug	Ciprofloxacin	−5.389	ASN 86, GLY 182
*Candida albicans* (CYP51, Oxidoreductase, Ergosterol synthesis)	Untreated cells extract	9-Octadecenoic acid (Z)	−1.539	TYR 505
	Treated cells extract	9-Octadecenoic acid (Z)	−1.539	TYR 505
	Untreated filtrate extract	9-Octadecenoic acid (Z)	−1.539	TYR 505
	Treated filtrate extract	Diisooctyl phthalate	−4.767	TYR 64
	Standard drug	Nystatin	−5.400	ASP 116

### Computational ADME profiling of bioactive metabolites identified in *C. hydrophila*: an in silico pharmacokinetic assessment

In silico ADME profiling was undertaken to assess the drug-likeness of the most active identified compounds (Table 5). All examined compounds stayed within acceptable limits of molecular weight according to Lipinski’s Rule of Five. They had high predicted human oral absorption (96–100%) with acceptable lipophilicity, although the predicted aqueous solubility was low. All compounds had low predicted blood-brain barrier penetration, which is a beneficial property of non-CNS-targeted antimicrobial agents.

**Table 5:** Pharmacokinetic predictions of selected metabolites based on QikProp ADME analysis

Compound	MW	Hydrogen bond donor	Hydrogen bond acceptor	SASA	QPlogPo/w	QPlogBB	QPlogS	% Human oral absorption
Diisooctyl phthalate	390.562	0	4	872.213	7.109	−1.121	−8.372	100.00
Hexadecanoic acid, TMS derivative	328.609	0	2	750.619	6.868	−0.714	−6.952	100.00
Palmitoleic acid, TMS derivative	327.557	0	2	740.479	6.723	−0.7	−6.8	98.05
Oleic acid (Z)-, TMS derivative	355.603	0	2	760.147	6.947	−0.9	−7.1	100.00
9-Octadecenoic acid (Z)	282.465	1	2	652.531	5.740	−1.073	−4.998	96.008
Drug-likeness criteria	< 500	≤ 5	≤ 10	300–1000	−2.0 to 6.5	−3.0 to 1.2	−6.5 to 0.5	> 80% is high

### Gene interaction analysis for targeting bacterial cell wall and candida *Candida* ergosterol pathways: a computational approach

**Fig. 12:**
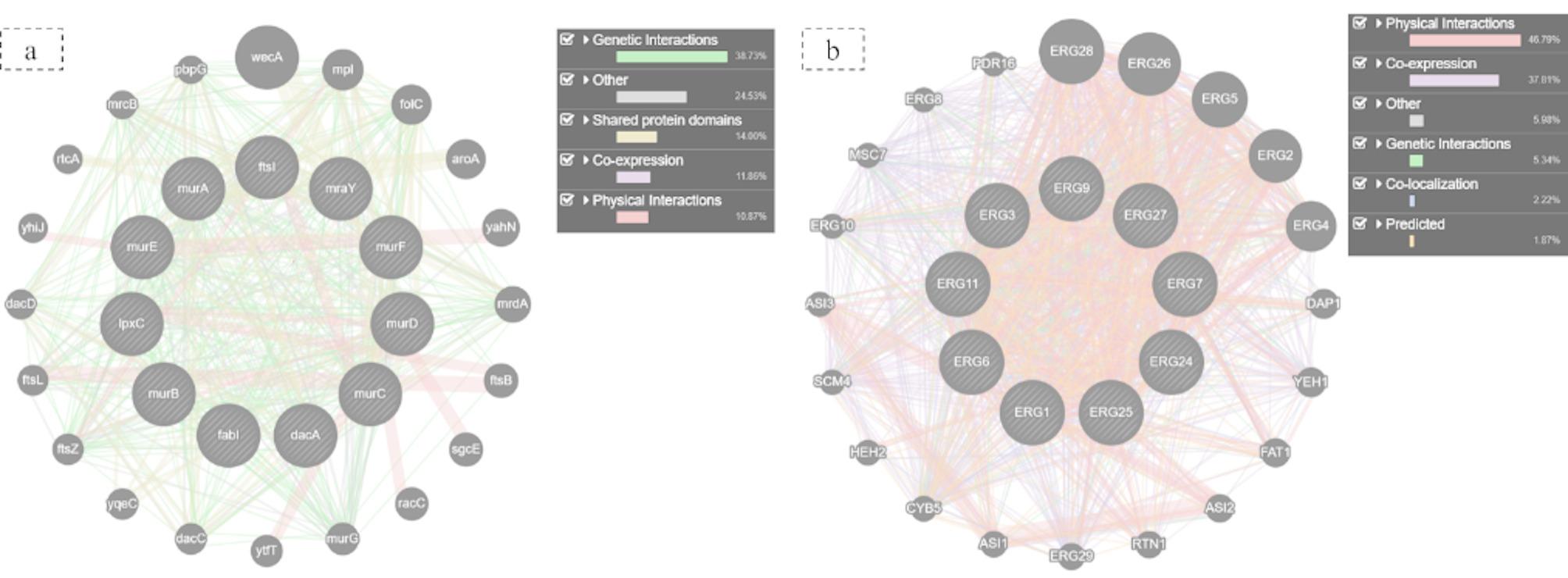
(**a**) gene interaction network of bacterial cell wall and membrane integrity targets and (**b**) gene association network of *C. albicans* ergosterol biosynthesis pathway

GeneMANIA was utilized for the analysis of the gene regulatory networks of the microbial targets. For the bacterial pathogens, the analysis showed very dense networks of genes linked to pathways for important building blocks for cell wall and membrane synthesis. For *C. albicans* there was a strong association among candidate ergosterol biosynthesis genes (mainly ERG11, ERG3, and ERG6) which validates their importance in maintaining the integrity of fungal membranes (Fig. 12).

## Discussion

The epigenetic modification of *C. hydrophila* by 5-azacytidine resulted in a number of substantive and complex changes, presenting very strong evidence of this strategy for natural product discovery. One of the main strengths of this study is the empirical validation that mechanistically links the use of this epigenetic trigger with the observed effects. This discussion will examine these interconnected outcomes from the initial molecular trigger to the final functional outcomes in order to provide a comprehensive overview of the profound impact of the intervention. Direct molecular evidence of demethylation and genomic effects was ultimately the foundational basis of this investigation. FTIR analysis exhibited the absence of spectrally significant peaks at 1420 and 1453 cm^−1^ in the samples exposed to 5-azacytidine. The C-H deformation and base stacking interactions in the B-form of DNA are represented by a 1420 cm^−1^ band. It is specifically linked to the deoxyribose C-H and sugar pucker conformation. The presence of this band suggests the normal B-form DNA structure. The CH_2_ scissoring vibrations in the deoxyribose sugar are represented by the 1453 cm^−1^ band [56, 57]. In spectroscopic investigations of DNA structure, these scissoring vibrations are crucial because they encode information regarding the dynamics and conformation of the DNA component [58, 59]. Variations in these vibrations may suggest modifications to the structure of DNA or interactions with other molecules, such as methyl groups [57]. This data provided direct molecular evidence of the strong epigenetic regulatory action of 5-azacytidine acting on *C. hydrophila*.

The ISSR profiling revealed a genomic modification of the 5-azacytidine mutated and non-mutated DNA. Nevertheless, it is widely recognized that DNA methylation does not influence the PCR amplification of target genes [61]. The absence or the emergence of new bands in the ISSR profiling may be a consequence of a varying primer binding affinity due to a demethylation event coincident with the aza treatment. Methylated cytosines exhibit distinct hydrogen bonding properties in comparison to unmethylated cytosines, which may result in a decrease in the efficiency of primer annealing if methylation sites are present in primer binding regions [60, 61]. The increase in marker production using the ISSR-5 primer possibly indicates the ability to employ this region as target methylation sensitive sequence in this organism [37]. In addition, the comet assay determined that there was no relative cytotoxic capacity similar to Xue et al. (2023) [7] yet produced a significant increase in DNA damage in susceptible cells. Demethylated DNA resulting from 5-azacytidine treatment is frequently more susceptible to breaks than methylated DNA. The chromatin structure of the methylated DNA is more compact, which reduces its susceptibility to detrimental agents like nucleases [62]. The present findings may be attributed to a decrease in DNA stability as a result of demethylation upon 5-azacytidine treatment, which altered the chemical properties of the DNA.

The validated epigenetic trigger resulted in significant changes in the metabolic output of *C. hydrophila*. GC-MS analysis of the extracts reveals significant differences in the metabolite profiles of the treated and untreated extracts, providing clear evidence for the activation of cryptic biosynthetic pathways, which is our primary goal through the use of epigenetic modifiers, as shown by Bind et al. (2022) [38]. Most notably, the treatment induced the production of entirely new compounds that were absent in the control, including 2-acetyl-3-(2-cinnamido)ethyl-7-methoxyindole and diisooctyl phthalate. In contrast, compounds found in the untreated samples, such as 9,12-octadecadienoic acid (Z, Z), have been previously indicated to possess antibacterial activities [39]. This outcome is consistent with published reports showing that demethylation can both induce new biosynthetic pathways and suppress specific metabolite(s) depending on strain, dose and the regulatory architecture of the biosynthetic gene clusters [40].

Conversely, the treatment induced a powerful antifungal activity against *C. albicans*. This aligns with studies on fungi like *Cochliobolus lunatus*, where 5-azacytidine treatment also increased the production of antifungal compounds [41]. This functional “trade-off” is a key finding and is similar to observations in *Shiraia bambusicola*, where treatment enhanced antifungal perylenequinone production while suppressing other metabolites [42]. This indicates that epigenetic modulation can effectively alter tumuli fungal metabolism in favor of any desired bioactivity [43, 44]. Changes were also observed in the secretome, where the treated filtrate extract had altered activity; again, indicating additional metabolite transportation pathways were affected and that 5-azacytidine had effects on the meta-solumon biotic process not just the intracellular biosynthesis [45–47].

The combination of our computational and bioinformatics-driven approach results in an in-depth mechanistic hypothesis to account for these experimental observations. The predicted down regulation of Pks1 promoter activities coincides with the observed loss of antibacterial activity as polyketides are prominent sources of fungal antimicrobials. As seen with several examples indicating a reversal of activation from demethylation are commonplace with different biosynthetic clusters exhibiting contrasting behaviour to the epigenetic alteration [48].

The predicted increase in promoter activity for Fas1 matches well with the increases in lipid-based metabolites, such as oleic and palmitoleic acids, evidenced in the GC-MS data. These fatty acids have been shown to compromise fungal membrane integrity [49] and are found to correspond with greater antifungal activity of epigenetically altered fungi [50]. An increase in transcriptional activation due to hypomethylation of a promoter is commonly observed in fungi, particularly with genes associated with lipid metabolism [51]. The predictive KEGG pathway analysis is in line with the conclusion of metabolic programming shift, as that type of pathway analysis represents such a shift from terpenoid biosynthesis to phenylpropanoid biosynthesis seen in other fungi, like Podospora anserina, that undergo epigenetic control [52].

Molecular docking analysis opposed to relevant enzymes from these validated pathways supports our experimental hypothesis. Metabolites from treated extracts, particularly diisooctyl phthalate, have shown substantial binding affinity improvement for the vital fungal enzyme CYP51 in *C. albicans*. This gives plausibility to the molecular explanation of this new strong antifungal activity. Additionally, the ADME profiling of these compounds showed drug-like properties as indicated by the favorable predicted drug-like properties, including a high intestinal absorption ability, low predicted CNS toxicity categorized as possible, and plausible compounds for scaffold development for pharmaceuticals [52].

To investigate these results further at a molecular level we selected protein targets for docking from essential microbial pathways. GeneMANIA network analysis indicated bacterial cell wall synthesis and fungal ergosterol biosynthesis pathways had a low amount of nonessential genes and were very densely connected, suggesting that the genes were essential since they are widely conserved and interconnected pathways that are targeted by a high number of successful antibiotics and antifungals [53, 54].

The present research successfully illustrates that 5-azacytidine mediated epigenetic modulation is an effective way to access the dormant biosynthetic capacity of the water fungus *C. hydrophila*. The most important aspect of this work was not simply an increase in metabolite yield but actually tailoring its metabolism and leading to a shift in function from an antibacterial to a potent antifungal potency. This study provides the first evidence of the induced production of novel compounds by this organism. Thus, the integrated genomics, metabolomics, and computational approaches utilized in this research provides an important proof-of-concept supporting the use of epigenetic means to screen the under-explored fungal niches mentioned in the introduction of this study for bioactive compounds and drug leads.

This research represents a strong proof-of-concept for future avenues of investigation. The gene expression analysis presented in this study was based on a predictive in silico model. A primary future investigative direction will be to empirically validate the predicted changes with wet-lab methods such as qRT-PCR. Additionally, a sustainability-oriented approach in this area is the ultimate connection of every new compound to its corresponding silent biosynthetic gene cluster (BGC). This step would involve whole-genome sequencing and functional genomic work, and our study contributes to the groundwork. Finally, the survivability of *C. hydrophila* subsequent to the metabolite production process is suggestive of innate self-resistance, which is worth investigating in future research for possible autotoxicity protective mechanisms.

## Supplementary Information

Below is the link to the electronic supplementary material.

Supplementary Material 1

## Data Availability

The datasets generated and/or analyzed during the current study are available in the NCBI repository, with accession number MK387081.
