# Supplementary material for: Epigenetic modulation of Ceratorhiza hydrophila by 5-azacytidine enhances antifungal metabolite production: insights from antimicrobial, metabolic, genomic and computational analyses
Source: BMC Microbiol. 2025 Sep 9;25:574. doi: 10.1186/s12866-025-04330-8 (PMC12418614; doi:10.1186/s12866-025-04330-8)
Supplement: Supplementary file 1 — Supplementary Material 1 [file 12866_2025_4330_MOESM1_ESM.docx]

**Supplementary material**


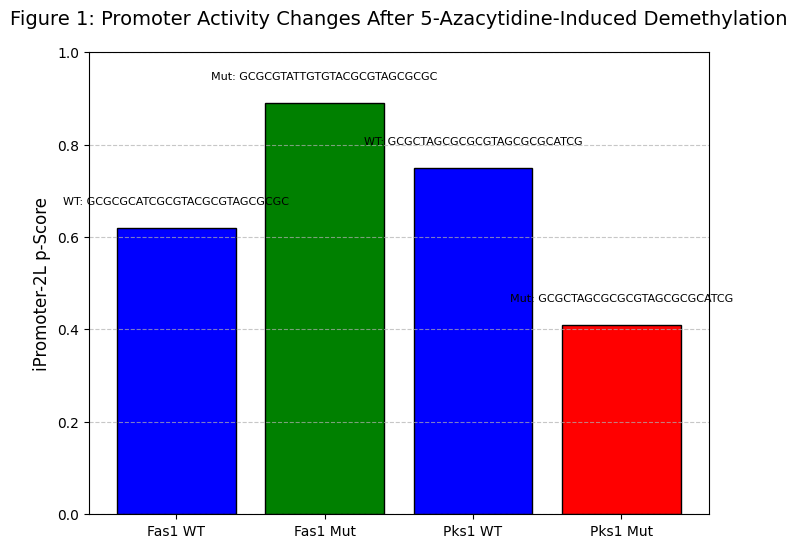


**Fig. S1.** Promoter activity (iPromoter-2L p-score) of *Fas1* and *Pks1* before (WT) and after (Mut) simulated demethylation, showing an increase for *Fas1* (0.62 to 0.89) and a decrease for *Pks1*  (0.75 to 0.41)


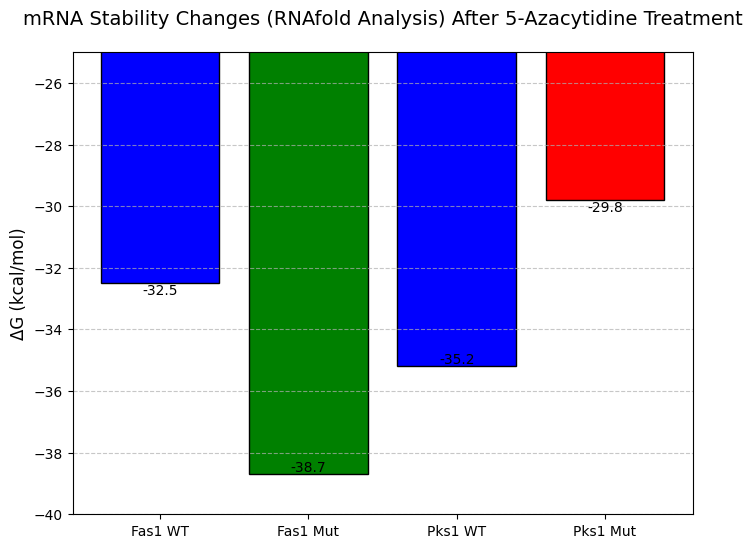


**Fig. S2.** mRNA stability (ΔG in kcal/mol) of *Fas1* and *Pks1* before (WT) and after (Mut) simulated demethylation, showing increased stability for *Fas1* (-32.5 to -38.7 kcal/mol) and decreased stability for *Pks1* (-35.2 to -29.8 kcal/mol)
